# Human bone marrow contains high levels of extracellular vesicles with a tissue-specific subtype distribution

**DOI:** 10.1371/journal.pone.0207950

**Published:** 2018-12-06

**Authors:** Andreas Rank, Rienk Nieuwland, Anton Köhler, Cordula Franz, Johanna Waidhauser, Bettina Toth

**Affiliations:** 1 2. Medizinische Klinik, Klinikum Augsburg, Augsburg, Germany; 2 Laboratory of Experimental Clinical Chemistry, and Vesicle Observation Centre, Academic Medical Center, Amsterdam, The Netherlands; 3 Medizinische Klinik und Poliklinik I, Ludwig Maximilians-Universität München, München, Germany; 4 Department of Obstetrics and Gynecology, University of Aachen, Aachen, Germany; 5 Gynecological Endocrinology and Reproductive Medicine, Medical University Innsbruck, Innsbruck, Austria; Wake Forest Institute for Regenerative Medicine, UNITED STATES

## Abstract

**Introduction:**

Extracellular vesicles (EV) are shed from a broad variety of cells and play an important role in activation of coagulation, cell to cell interaction and transport of membrane components. They are usually measured as circulating EV in peripheral blood (PB) and other body fluids. However, little is known about the distribution, presence and impact of EV and their subpopulations in bone marrow (BM). In our study, we focused on the analysis of different EV subtypes in human BM as compared to EV subsets in PB.

**Methods:**

EV in BM and PB from 12 healthy stem cell donors were measured by flow-cytometry using Annexin V and cell-specific antibodies for hematopoietic stem cells, leucocytes, platelets, red blood cells, and endothelial cells. Additionally, concentrations of tissue factor-bearing EV were evaluated.

**Results:**

High numbers of total EV were present in BM (median value [25–75 percentile]: 14.8 x10^9^/l [8.5–19.3]). Non-significantly lower numbers of total EV were measured in PB (9.2 x10^9^/l [3.8–14.5]). However, distribuation of EV subtypes showed substantial differences between BM and PB: In PB, distribution of EV fractions was similar as previously described. Most EV originated from platelets (93.9%), and only few EV were derived from leucocytes (4.5%), erythrocytes (1.8%), endothelial cells (1.0%), and hematopoietic stem cells (0.7%). In contrast, major fractions of BM-EV were derived from red blood cells or erythropoietic cells (43.2%), followed by megacaryocytes / platelets (27.6%), and by leucocytes as well as their progenitor cells (25,7%); only low EV proportions originated from endothelial cells and hematopoietic stem cells (2.0% and 1.5%, respectively). Similar fractions of tissue factor—bearing EV were found in BM and PB (1.3% and 0.9%).

**Conculsion:**

Taken together, we describe EV numbers and their subtype distribution in the BM compartment for the first time. The tissue specific EV distribution reflects BM cell composition and favours the idea of a BM–PB barrier existing not only for cells, but also for EV.

## Introduction

Initially described as cell dust in 1946 [1], extracellular vesicles (EV) are membrane vesicles released from different cell types. Circulating EV in blood are derived from all vascular and blood cells [2] and differ in size from 0,1 to 1 μm. They contain a mixture of lipids, proteins and surface markers, which reflects the composition of their cellular origin [3]. EV formation is triggered by apoptosis and cell activation and is known to have various physiological and pathological functions such as transport of membrane components, activation of inflammation and stimulation of coagulation [4]. It has become more and more evident that circulating EV can serve as surrogate markers for diseases like diabetes [5], hypertension [6], arterial thrombosis [7], or sepsis [8]. Moreover, EV seem to be involved in oncogenesis, tumor progression, and pathogenesis of metastasis in cancer patients [9,10,11]. EV have been sucessfully utilized as diagnostic biomarkers in patients with changiocarcinoma, hepatocellular and lung cancers [12,13,14]. Furthermore, some cancer cells use EV for export of antineoplastic drugs mediating drug resistenance [15].

Recently, EV have also been detected in other tissues and body fluids. For example, high levels of EV were detected in cerebrospinal fluid in patients after brain injury [16]. György et al. found EV in joint fluid in different concentrations and membrane patterns, dependent on the underlying arthropathy [17].

However, little is known about EV in the BM compartment. BM is the major hematopoietic organ and a primary lymphoid tissue, responsible for the production and maturation of erythrocytes, granulocytes, monocytes, lymphocytes and platelets. There is strict control of cell migration from BM into PB and vice versa. The endothelium of BM sinuses is a continuous layer which contains a selective cellular transport system that serves as the marrow-blood barrier. Our data suggest that this barrier is relevant also for EV.

The aim of this study was to detect frequency and composition of EVs in the BM compartment of healthy individuals in comparison to the corresponding EV fraction in PB.

## Patients and methods

### Study population and trial design

12 healthy hematopoetic stem cells donors were recruited (6 women, 6 men, median age 42 years (range: 21–56 years) between April 2011 and January 2012. All of them donated BM for family members undergoing hematopoietic stem cell transplantation. Donors were healthy without diagnosis of chronic disease (especially hepatitis or HIV were excluded). PB counts were normal for each donor. PB and BM specimens were taken during routine sampling.

The Human Investigation Review Board of the Ludwig-Maximilian-University Munich approved the study. Signed written informed consent for the study was obtained from all donors.

### Blood sampling and assessment

PB samples (5ml) were taken from donors via hollow needle (20 gauge) without swelling to avoid platelet activation. Paired 5 ml samples of aspirated BM blood were harvested by low negative pressure using a large-core Jamshidi needle (11 gauge). Samples from both compartments were centrifuged for 20 minutes at 1,550 × g within 15 minutes after sampling, plamsa fraction was kept in liquid nitrogen for 15 minutes to stabilize the samples and avoid uncontrolled EV formation. Samples were then stored at– 80°C.

### Isolation of extracellular vesicles

Samples were thawed carefully over approximately one hour and isolated regarding to the description by Berckmans RJ et al. [18]: after centrifugation of 250 μl plasma for 30 minutes with a centrifugal force of 18,890 x g, 225 μl plasma were removed. EV were resuspended after addition of 225 μl of citrate buffer (3,2% tri-natrium-citrate) and centrifuged again for 30 minutes with 18,890 x g. After removal of 225 μl of EV free supernatant, 75 μl citrate buffer was added and the EV pellet was resuspended again. For detection of EV bearing cell-specific antigens, 5 μl of suspension were incubated for 15 minutes with 5 μl of APC-labeled annexin V (IQ products, Groningen, The Netherlands) in the dark at room temperature together with a cell-specific antibody for double staining: PE-labeled anti-CD45 (BD Pharmingen, San Diego, USA) for leucocyte-derived EV, FITC-labeled anti-CD235a (DakoCytomation, Denmark) for erythrocyte-derived EV, PE-labeled anti-CD34 (BD Pharmingen, San Diego, USA) for stem cell-derived EV, PE-labeled anti-CD62E (BD Pharmingen, San Diego, USA) for endothelium-derived EV or PE-labeled anti-CD142 (BD Pharmingen, San Diego, USA) for EV carrying tissue factor. For detection of platelet-derived EV, triple-staining was used: APC-labeled annexin V (IQ products, Groningen, The Netherlands) together with FITC-labeled anti-CD61 (DakoCytomation, Denmark) as common platelet marker and, additionally, platelet activation marker PE-labeled anti-CD62P (Immunotech, Marseille, France) or alternativly PE-labeled anti-CD63 (Immunotech, Marseille, France). The reaction was stopped with 900 μl of calcium buffer (2,5 mmol/l) except for the annexin V control, which was filled up with 900μl citrate-containing PBS. EV were resuspended before flow cytometry analysis.

### Analysis of EV

**Fig 1:**
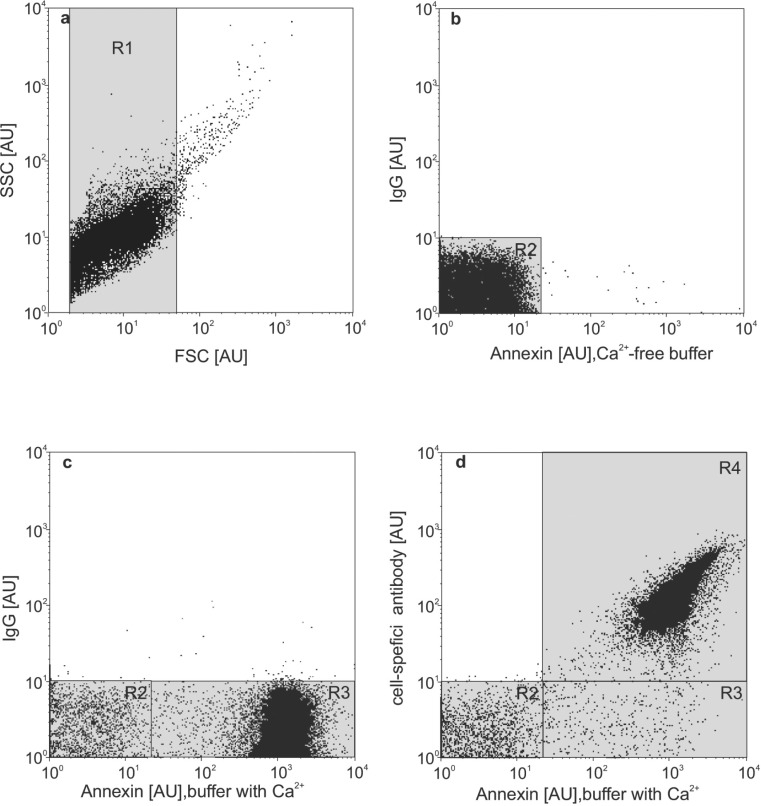
Gating strategy for identification of microparticle (EV). First, EV were identified by their dimension as described by Nieuwland et al. [19] (a). As negative control, EV were double stained with control antibody IgG plus annexin V in a Ca2+-free buffer (b). After addition of Ca2+, annexin V binds with high specificity and sensitivity to EV (c; R3). Double staining with annexin V and a cell-specific antibody identifies EV subpopulations (d; R4).

All samples were analysed with a FACScalibur flow cytometer (Becton Dickinson, Heidelberg, Germany) running the Cell Quest Software (Becton Dickinson, San Jose, CA, USA). The flow cytometer was run on high pressure. Forward (FSC) and sideward scatter (SSC) were set to logarithmic gain and cell derived EV were identified via simultaneous binding to annexin V and markers of EV origin cells. All samples were analysed for one minute. The volume analysed in one minute (V; approximately 50–80 μl) was determined each day before analysis by taking the weight of a sample (aqua dest) before and after analysis. The gating strategy for isolation of EV is shown in Fig 1. The number of EV/μl of plasma was determined by using following formula: EV/l = N x (100μl/5μl) x (950μl/V) x 10^6^/250μl) according to Berckmans RJ *et al* [11] (N = absolute number of EV determined by FACS analysis; 100μl = total volume of washed EV suspension; 5 μl = pellet used for analysis; 950μl = total volume before analysis (pellet + antibodies + buffer); 250μl = original volume of the sample before isolation of EV).

### Statistical analysis

Results are reported as median and interquartile range, if not stated otherwise. Variables were tested by the paired non-parametic Wilcoxon test, and Spearman test for correlation coefficients. Statistical analyses were performed using the Statistical Package for the Social Sciences SPSS for Windows 20.0 (SPSS Inc., Chicago, IL, USA). All p-values are two-sided, p-values <0.05 are considered significant. Due to the exploratory nature of our analyses, no adjustment for multiple testing was undertaken.

## Results

### Total EV

**Fig 2:**
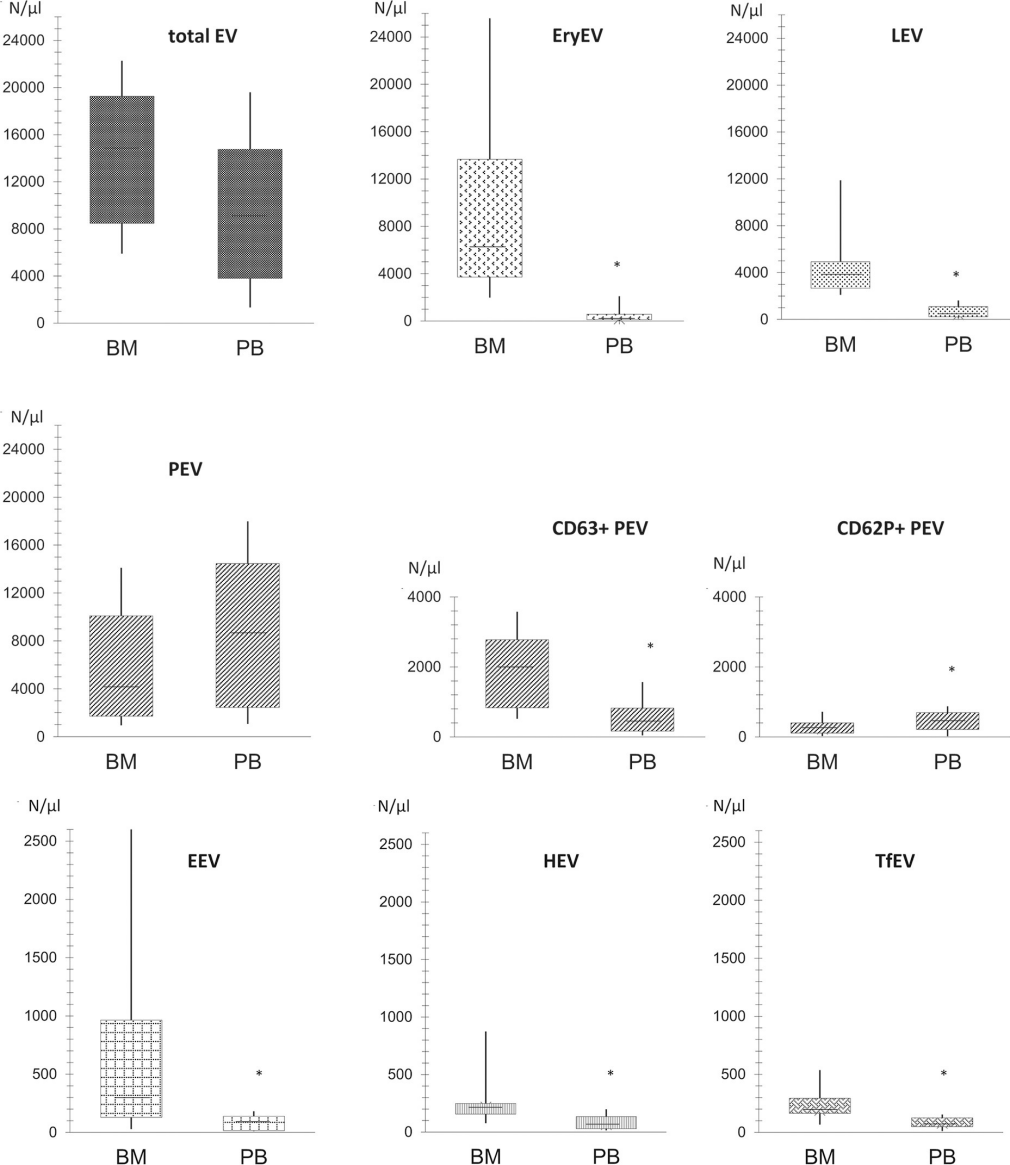
Total EV and their subsets in BM versus PB. X-axis: compartment of bone marrow (BM) versus compartment of peripheral blood (PB). Y-axis: Concentration of total EVs and respective subpopulations; EryEV: EV derived from erythrocytes or their progenitor cells, LEV: EV derived from leukocytes or their progenitor cells, PEV: EV derived from platelets or megakaroycytes, EEV: EV derived from endothelium cells, HEV: EV derived from hematopoetic stem cells, TfEV: EV bearing tissue factor. Level of significance p < 0.05.

In BM of healthy humans (BM-EV), a high total number of annexin V-binding EV was detected without reaching statistically significant differences compared to EV in PB (9.2 x10^9^/l [3.8–14.5] vs. 14.8 x10^9^/l [8.5–19.3], p = 0.060). Data of total EV and their subset distribuation are shown in Fig 2, exact values are given in supplement (S1 Table). No statistically significant correlations were found between BM-EV and PB-EV or their corresponding subpopulations. Pairwise data of total BM-EV versus PB-EV and their subtypes from each donor are also shown in supplement (S1 Fig).

### Erythrocyte-derived EV (EryEV)

EryEV were identified as CD235a positiv EV and represented the major sub-fraction (43.2%) of all detected EV in BM. In contrast, only a small portion of EryEV was present in PB (1.8%).

### Platelet-derived EV (PEV)

CD61-exposing PEV (27.6%) constituted the second largest EV-fraction in BM. In PB, PEV represented the major subgroup (93.9%) of all measured circulating EV. The portion of activation marker CD63—bearing PEV was nearly half in the BM compartment; in contrast, only a small fraction of PEV in PB was found to be CD63 positive. However, the second activation maker CD62P was discovered on approximately 5% of PEV in both compartments.

### Leucocyte-derived EV (LEV)

25.7% EV in BM were CD45 positive. In contrast, CD45-positive EV in PB were found only in 4.5%.

### Endothelial EV (EEV)

The proportion of CD62E-positive EEV was low in BM (2%) and even less in PB (1%).

### Haematopoetic stem cell derived EV (HEV)

CD34 positive HEV were detected with low frequency in PB (0.7%) and with higher frequency in BM (1.5%).

### Tissue factor bearing EV (TfEV)

1.3% of total EV in BM were CD142 positive, 0.9% in PB.

## Discussion

Total EV numbers in BM was higher than in PB. This difference was not statistically significant most likely due to low sample size of 12 donors. EV mainly arise during cell activation or cell death and carry antigens from their cells of origin. Thus, it is not surprising to detect a considerable high count of EV in human BM, a compartment with high cell proliferation. One of the major findings of this study is the fundamentally different distribution of EV subtypes in BM compared to circulating EV in PB possibly reflecting different cell composition of these two compartments. Assuming that EV synthesis and function in BM is equal or just similar to PB, it is highly possible that EV play a pivotal role in cell to cell—communication. As EV contain parts of the outer membrane and the cytoplasm of their cells of origin, they may present a broad variety of cell adhesion and signalling molecules on their surface. Cells can even mediate intercellular communication by secreting EV enriched with different RNA species [20]. As we have found high levels of EV in BM, they might be of importance for regulation und interaction of hematopoiesis and immune functions inside BM. Furthermore, EV play a major role in activation of plasmatic coagulation by presenting a large activated phospholipid surface with binding sites for several different coagulation factors [21,22]. This important function becomes obvious in patients with Scott syndrome suffering from a haemophilia-like bleeding disorder due to a high-grade deficiency of platelet-derived EV [23]. Conversely, elevated levels of circulating EV are associated with thrombotic events [24]. Following these clinical observations, co-transfusion of EV together with stem cells during BM transplantation leads to a temporary increase of circulating EV in transplanted patients which is considered to be causative for venous thrombotic complications [25]. Particularly, a higher incidence of catheter associated thrombotic event is described during the first weeks of transplantation [26].

In our investigation, the largest subfraction of EV in BM was found to be derived from erythrocytes and further erythropoetic cells. This reflects the high abundance and proliferation / differentiation activity of erythroblastic cells. Erythropoesis occurs in distinct cellular units, called erythroblastic islands and seems to be accompanied by high rate of shedded EV carring CD279 on their surface. Hence, EV are involved in cell maturation of erytroid cells e.g. they are postulated to have an important function during terminal differentiation of reticulocytes into erythrocytes. EV and other microvesicles are thought to remove cellular molecules or organelles no longer required at this step of erythroid differentiation [27,28]. In contrast, we detected much lower EryEV levels in PB indicating that adult red blood cells release only a very small number of EV. This finding is in line with other reports demonstrating that mature erythrocytes shed almost no EV under physiological conditions. Also, patients with infections or even septic infections don´t exhibit elevated circulating EryEV numbers. Elevated EryEV levels have only been described under distinct medical conditions like in patients with sickle cell disease [29], in preeclamptic women [30], and in patients with acute graft-versus-host disease or with microangiopathy [31].

The second largest EV fraction in BM was CD61 positive. It may surprise that approximately one quarter of EV orginates from platelets whereas more than 90% of EV are derived from platelets in PB. This difference could be explained by the fact that megakaryopoiesis occurs adjacent to the sinus endothelium. Platelets and probably related CD61-positive EV are released together directly into the blood stream [32]. This might cause the relatively low levels of PEV in BM. However, our data would also support the hypothesis that at least a part of PEV in PB originate from megakaryocytes, as they have direct access to the blood stream and cannot be distinguished from EV derived from circulating platelets [33]. This, however, does not exclude that some CD61-positive EV are released from megakaryocytes. Only a low portion of PEV was positive for the activation marker CD62P in both investigated compartments. The other activation marker CD63 was detected on PEV at also similar low level in PB. Surprisingly, CD63 accounted for almost half of CD61-positive EV in BM. CD63 is a member of the tetraspanin family commonly used as a marker for strong platelet activation. It is expressed on the inner surface of platelet lysosomes. However, it is not confined to platelets: CD63 is present in late endosomes and lysosomes of other cell types and participates in a variety of cellular processes, like cell activation, adhesion, and differentiation. In late endosomes, CD63 is enriched on the intraluminal vesicles, which by activation are secreted through fusion with the plasma membrane [34, 35].

Another quarter of BM-EV belongs to cells from lymphopoiesis and myeloid cells. In PB, only a small number of EV was CD45 positive which could possibly be explained by the analysis of healthy donors in our study population. Elevated numbers of leucocyte derived EV can be mainly found after activation of white blood cells like described for example in patients with atherosclerosis [36] or with severe infection like Shiga-toxin mediated haemolytic uremic syndrome [37].

Endothelial derived EV represent only a small part of al EV. However, we measured nearly four times higher concentrations of this EV subtype in BM compared to PB, In PB, endothelial cell EV are commonly regarded a biological marker of endothelial activation or apoptosis [38]. In the Framingham Heart study circulating endothelium-derived microparticles were associated with cardiometabolic risk factors [39]. Furthermore, either elevated concentration or altered composition of this EV subtype could be found in a broad variety of diseases like vasculitis [40], acute coronary syndrom [41], mitral valve disease [42] or thrombotic thrombocytopenic purpura [43]. The relatively high EEV levels in BM of healthy donors might reflect the fact that endothelium is highly abundant and active in BM.

A small number of BM-EV orginates from CD34-positive hematopoietic stem cells. This EV subtype is even less frequent in PB. The source of circulating hematopoietic stem cell EV, however, is not clear: either they are released from circulating stem cells or they are able to penetrate the blood-marrow barrier.

Tissue factor (TF, CD142) is the major initiator of the extrinsic coagulation cascade and is mainly expressed on non-vascular cells like epidermis, renal glomeruli or smooth muscle cells. In PB, main reservoir of circulating TF is the pool of CD142-positive EV [44]. Enhanced levels of TF-carrying EV have been observed in cancer patients, in patients with unstable angina [45], and in patients with severe infections/sepsis. In our study, TF-positive EV were measured at low frequency in PB of healthy humans. TF-EV abundance was slightly higher in BM.

These presenting findings of our study are restricted by some limitations. First, the number of investigated individuals is small depending on the fact that our investigation was planned as a basic research project to provide evidence of the existence of EV in BM. Furthermore, we were not able to correlate BM-EV with counts of their mother cells in BM because, from an and ethical point of view, it is not possible to take a bone marrow biopsy from a healthy donor for histological analyses not required for the stem cell donation itself. Lastly, our analyses were restricted to an EV size of 500 nm due to the technique of flow cytometry. Recent published data from the ISTH-SSC-VB Working group [46] revealed and discussed this limitation by comparing 46 different types of flow cytometer. Thus, exosomes and other smaller EV are not detectable by the applied method of flow cytometry.

In summary, our data firstly demonstrate a high level of EV in BM with a fundamental different subgroup composition compared to PB. This finding suggests that the physiological barrier between BM and PB exists not only for cells but also for EV.

## Supporting information

S1 Table Numbers of EV in bone marrow vs. peripheral blood. Numbers are given as mean values and interquartile range. EryEV: EV from erythrocytes / erythropoetic cells. PEV: EV from megacaryocytes / platelets. LEV: EV from leucocytes / leucocytic progenitor cells. EEV: EV from endothelial cells. HEV: EV from hematopoetic stem cells. TfEV: tissue factor—bearing EV.
(DOCX)

S2 Table Data file. Measured EV data from al donors in N/μl.
(PDF)

S1 Fig Total EV and their subsets. Data presented as EV concentrations in bone marrow versus peripheral blood from each donor. EryEV: EV derived from erythrocytes or their progenitor cells, LEV: EV derived from leukocytes or their progenitor cells, PEV: EV derived from platelets or megakaroycytes, EEV: EV derived from endothelium cells, HEV: EV derived from hematopoetic stem cells, TfEV: EV bearing tissue factor.
(TIF)
