# Supplementary material for: Human bone marrow contains high levels of extracellular vesicles with a tissue-specific subtype distribution
Source: PLoS One. 2018 Dec 6;13(12):e0207950. doi: 10.1371/journal.pone.0207950 (PMC6283575; doi:10.1371/journal.pone.0207950)
Supplement: S1 Table — Numbers are given as mean values and interquartile range. EryEV: EV from erythrocytes / erythropoetic cells. PEV: EV from megacaryocytes / platelets. LEV: EV from leucocytes / leucocytic progenitor cells. EEV: EV from endothelial cells. HEV: EV from hematopoetic stem cells. TfEV: tissue factor—bearing EV. (DOCX) [file pone.0207950.s001.docx]

|  | ***bone marrow*** |  | ***peripheral blood*** |
| --- | --- | --- | --- |
| *N =* | *12* |  | *12* |
|  |  |  |  |
| ***Annexin V+ EV****(total EV)* *[x10^6^/l]* | **14792** (8466-19260) | *p=0.060* | **9211** (3806-14523) |
| ***CD235a+ EV*** *(EryEV) [x10^6^/l]* | **6384** (3721-13654) | *p=0.002* | **166** (104-575) |
| *% of total EV* | 43.2 |  | 1.8 |
|  |  |  |  |
| ***CD61+ EV*** *(PEV) [x10^6^/l]* | **4082** (1717-10087) | *p=0.084* | **8651** (2449-14452) |
| *% of total EV* | 27.6 |  | 93.9 |
|  |  |  |  |
| ***CD63+ PEV*** *[x10^6^/l]* | 1891 (892-5883) | *p=0.003* | 521 (165-930) |
| *% of PEV* | 46.3 |  | 6.0 |
|  |  |  |  |
| ***CD62P+ PEV*** *[x10^6^/l]* | 228 (109-401) | *p=0.049* | 452 (214-691) |
| *% of PEV* | 5.6 |  | 5.2 |
|  |  |  |  |
| ***CD45+ EV*** *(LEV) [x10^6^/l]* | **3798** (2675-4923) | *p=0.002* | **414** (221-1099) |
| *% of total EV* | 25.7 |  | 4.5 |
|  |  |  |  |
| ***CD62E+ EV*** *(EEV) [x10^6^/l]* | **294** (129-964) | *p=0.006* | **89** (15-138) |
| *% of total EV* | 2.0 |  | 1.0 |
|  |  |  |  |
| ***CD34+ EV*** *(HEV)* *[x10^6^/l]* | **216** (165-248) | *p=0.002* | **65** (30-134) |
| *% of total EV* | 1.5 |  | 0.7 |
|  |  |  |  |
| ***CD142+ EV*** *(TfEV) [x10^6^/l]* | **190** (164-293) | *p=0.004* | **79** (49-124) |
| *% of total EV* | 1.3 |  | 0.9 |

**Table 1: Numbers of EV in bone marrow vs. peripheral blood**

Numbers are given as mean values and interquartile range. EryEV: EV from erythrocytes / erythropoetic cells. PEV: EV from megacaryocytes / platelets. LEV: EV from leucocytes / leucocytic progenitor cells. EEV: EV from endothelial cells. HEV: EV from hematopoetic stem cells. TfEV: tissue factor - bearing EV.
