# Supplementary material for: Human bone marrow contains high levels of extracellular vesicles with a tissue-specific subtype distribution
Source: PLoS One. 2018 Dec 6;13(12):e0207950. doi: 10.1371/journal.pone.0207950 (PMC6283575; doi:10.1371/journal.pone.0207950)
Supplement: S2 Table — Measured EV data from al donors in N/μl. (PDF) [file pone.0207950.s002.pdf]

Pat_Nr	total Ev in BM	total EV in PB	PEV in BM	PEV in PB	CD62P+ EV in BM	CD62P+ EV in PB	CD63+ PEV in BM	CD63+ PEV in PB
1	5900	10780	1624	10713	163	790	1135	858
2	16642	15043	11207	14737	628	472	2001	455
3	6105	10079	1474	8848	53	546	733	901
4	14282	13879	1996	13600	152	414	1328	414
5	20969	17138	14111	16803	715	876	3576	1565
6	14681	19606	10663	17991	228	433	2211	711
7	22285	5374	8360	4930	378	194	2472	244
8	18661	6196	3272	6613	295	740	1781	629
9	9094	2293	2787	1622		275	614	138
10	8257	1341	948	1074	25	19	519	45
11	14902	3284	4892	1209	109	130	2874	78
12	19459	8344	7682	8455	401	500	3428	587

Pat_Nr	EEV in BM	EEV in PB	TfEV in BM	Tf EV in PB	EryEV in BM	EryEV in PB	LEV in BM	LEV in PB	HEV in BM	HEV in PB
1	386	138	171	78	4254	76	3474	214	148	15
2	33	79	196	110	5205	39	2667	325	182	144
3	331	175	68	84	2057	1160	2630	1629	78	26
4	123	92	240	129	14063	104	2112	643	396	104
5	1428	95	184	144	5957	105	4739	1210	229	158
6	486	182	164	154	6811	580	4124	1295	201	198
7	188	78	473	80	20061	135	3641	502	90	40
8	1123	89	537	55	25586	296	4984	765	255	63
9	256	15	164	19	3544	559	2699	243	227	67
10	30		99	11	1992	196	3956	210	226	39
11	2613	10	311	48	10983	2095	11880	162	875	85
12	148	13	212	60	12429	110	6361	272	207	26
