# Supplementary figures and images for: Human bone marrow contains high levels of extracellular vesicles with a tissue-specific subtype distribution
Source: PLoS One. 2018 Dec 6;13(12):e0207950. doi: 10.1371/journal.pone.0207950 (PMC6283575; doi:10.1371/journal.pone.0207950)

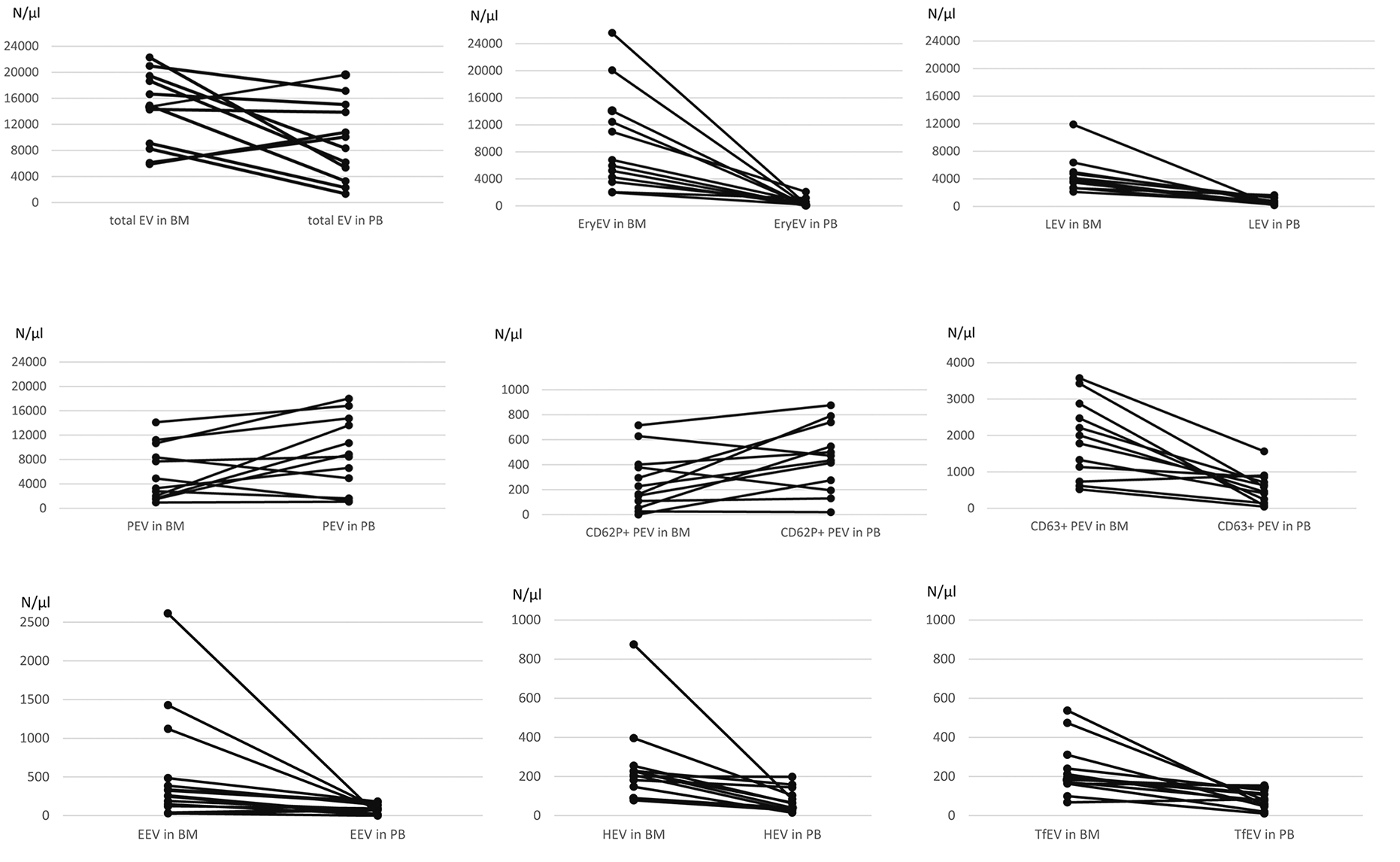

Supplement: S1 Fig — Data presented as EV concentrations in bone marrow versus peripheral blood from each donor. EryEV: EV derived from erythrocytes or their progenitor cells, LEV: EV derived from leukocytes or their progenitor cells, PEV: EV derived from platelets or megakaroycytes, EEV: EV derived from endothelium cells, HEV: EV derived from hematopoetic stem cells, TfEV: EV bearing tissue factor. (TIF) [file pone.0207950.s003.tif]
